# Increased Glutamine Consumption in Cisplatin-Resistant Cells Has a Negative Impact on Cell Growth

**DOI:** 10.1038/s41598-018-21831-x

**Published:** 2018-03-06

**Authors:** Guihua Duan, Mengyue Shi, Lijuan Xie, Mingcui Xu, Yun Wang, Hongli Yan, Yuzheng Zhuge, Xiaoping Zou

**Affiliations:** 1 Department of Gastroenterology, Drum Tower Hospital, Medical School of Nanjing University, Nanjing, 210008 China; 2 Department of Gastroenterology, The First People’s Hospital of Yunnan Province, The Affiliated Hospital of Kunming University of Science and Technology, Kunming, 650032 China; 3 Department of Special Medical Treatment, First Affiliated Hospital of Kunming Medical University, Kunming, 650332 China; 4 Department of Laboratory Medicine, Changhai Hospital, Second Military Medical University, Shanghai, 200433 China

## Abstract

The emergence of drug-resistant subclones remains the primary reason for tumor treatment failure. Some theories suggest that drug-resistant cell growth can be suppressed by drug-sensitive cells because resistant cells are less fit than sensitive cells in the absence of drug. We investigated fitness differences and their underlying mechanisms in cisplatin (ddp)-resistant cells and parental cells. We found that glutamine (Gln) consumption was substantially higher in ddp-resistant cells than that in sensitive cells, indicating that significantly fewer ddp-resistant cells than sensitive cells could be generated under the same Gln conditions. Interestingly, the antioxidant capacity of ddp-resistant cells was also significantly enhanced and was directly related to the presence of Gln. Then, we found that enhanced antioxidant capacity was sustained by accelerated Gln catabolism in resistant cells through oncogenic KRAS. Further analysis indicated that rapid Gln catabolism directly mediated ddp resistance through enhanced antioxidant capacity, but the maximum number of resistant cells that could be produced with the same amount of Gln was significantly reduced due to increased Gln catabolism. Collectively, our study revealed that rapid Gln catabolism provided ddp-resistant cells with the ability to tolerate cytotoxic treatment but also hindered the growth of ddp-resistant cells due to excessive Gln consumption.

## Introduction

In the tumor microenvironment, a sophisticated ecological system, genetically or epigenetically distinct subclones can intermingle or be spatially separated, and this subclonal architecture changes dynamically during tumor progression^1,2^. This intratumor heterogeneity enables subclonal evolution under selective pressure during cytotoxic treatment^3–5^. Numerous studies have demonstrated the presence of drug-resistant cells prior to treatment initiation^6–8^, and cytotoxic therapy kills a large number of tumor cells while providing resistant cells the opportunity to rapidly expand^9–11^. However, some theories suggest that drug resistance mechanisms require the consumption of additional resources for proliferation, and consequently, resistant cells may be less fit than sensitive cells in the absence of the drug^10,12^. Therefore, certain computational models and experiments have demonstrated that patient survival time could be prolonged by exploiting the competition between drug-sensitive and drug-resistant cells^13–18^ compared with tumor eradication using traditional continuous treatment. However, the underlying mechanisms by which drug resistance impacts the fitness of resistant cells are poorly understood.

Platinum-based drugs such as cisplatin (ddp) are commonly used in the treatment of many advanced cancers and often lead to initial therapeutic success associated with partial responses or disease stabilization, but eventually, chemoresistant subclones emerge and lead to therapeutic failure^19^. It has been demonstrated that ddp-resistant cells are less fit than ddp-sensitive cells^18^, but the underlying mechanisms are not known.

Cellular reactive oxygen species (ROS) are primarily generated through mitochondrial oxidative phosphorylation and can also be generated during the cellular response to exogenous substances^20^. Maintenance of redox homeostasis is important for cell growth and survival. The interaction between ddp and reduced glutathione (GSH) in the cytoplasm disrupts the cellular redox balance, and increased ROS can promote ddp-induced DNA damage^19^. A recent study indicated that while most cells use Gln to fuel the tricarboxylic acid (TCA) cycle, pancreatic cancer depends on a distinct pathway in which glutamine (Gln) can be used to maintain the cellular redox state by metabolic reprogramming mediated by oncogenic KRAS^21^. Gln is a key substrate required for the metabolism of proliferating cells because it serves as a carbon source to fuel the TCA cycle and transfers nitrogen for the biosynthesis of proteins, nucleotides and hexosamine^22,23^. This process implies that metabolic Gln reprogramming mediated by oncogenic KRAS may be related to the ddp-resistance mechanism in certain cell types.

In this study, we revealed that ddp-resistant cells sustained enhanced antioxidant ability to mediate ddp resistance via rapid Gln catabolism and that this metabolic reprogramming was mediated by oncogenic KRAS. Therefore, resistant cells consumed substantially more Gln than sensitive cells to support growth. However, rapid Gln catabolism is unnecessary and can even be a burden to the growth of ddp-resistant cells in the absence of ddp.

## Results

### Ddp-resistant cells consume significantly more Gln during growth

**Figure 1:**
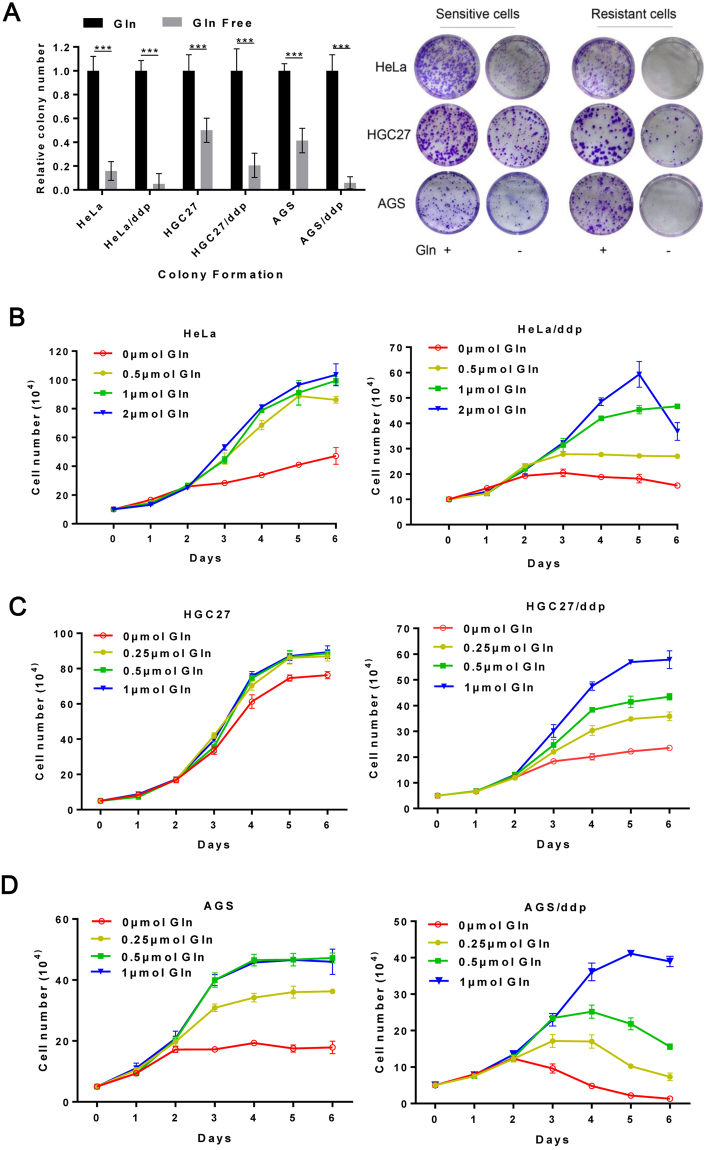
The number of ddp-resistant cells increased with increased total Gln. (**A**) Relative clonogenic growth of HeLa, HeLa/ddp, HGC27, HGC27/ddp, AGS and AGS/ddp cells under normal (2 mM Gln) or Gln-free conditions; the medium was replaced the following day with normal or Gln-free medium, and then the medium was not altered throughout the course of the experiment. (**B**,**C** and **D**) Growth of HeLa, HeLa/ddp, HGC27, HGC27/ddp, AGS and AGS/ddp cells in different amounts of Gln; the medium was replaced the following day with media containing different amounts of Gln, and then the media was not altered throughout the course of the experiment. The error bars represent the s.d. of triplicate wells from a representative experiment.

**Figure 2:**
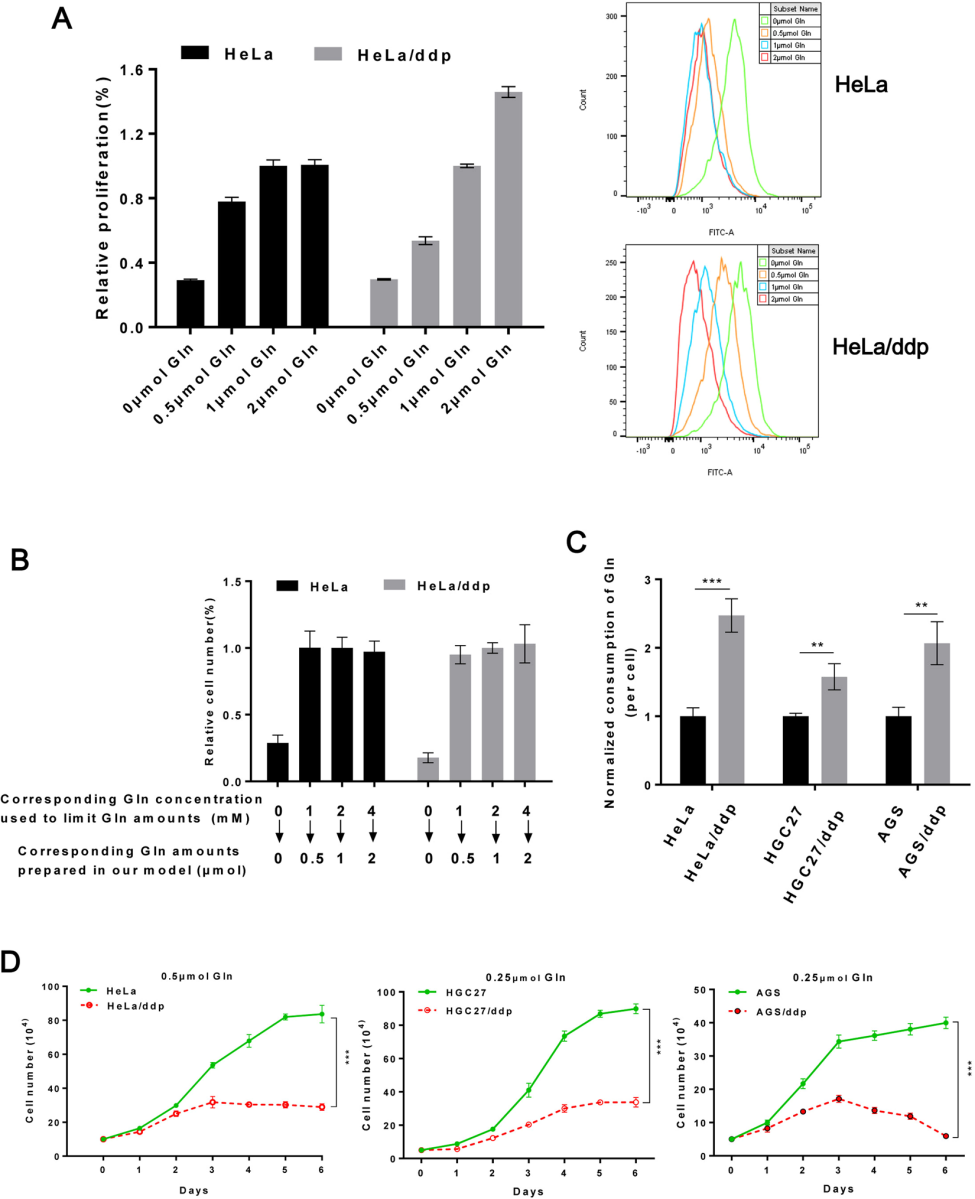
Resistant cells consumed more Gln than sensitive cells to sustain growth. (**A**) Relative proliferation of HeLa and HeLa/ddp cells cultured with different amounts of Gln determined by CFSE assay; the medium was replaced the following day with media containing different amounts of Gln, and then the media were not altered throughout the course of the experiment. (**B**) Proliferation of HeLa and HeLa/ddp cells in media with different Gln concentrations; media were replaced every day to maintain sufficient Gln supplementation. (**C**) Quantification of Gln consumption in three ddp-resistant cell lines and their parental cell lines 24 hours after medium replacement. (**D**) Growth of sensitive and ddp-resistant cells in the same amount of Gln; the medium was replaced the following day with media containing different amount of Gln, and then the media were not altered throughout the course of the experiment. The error bars represent the s.d. of triplicate wells of a representative experiment.

Glucose, fatty acids and Gln are three primary substances used for cellular energy metabolism^24^, and Gln is the most abundant amino acid in the human body. No previous studies have investigated the relationship between ddp resistance and Gln consumption. In our study, a colony formation assay first confirmed that Gln was an important factor for the growth of resistant and sensitive cell populations (Fig. 1A). Next, we observed that with increased total Gln (1 and 2 μmol of Gln for HeLa and HeLa/ddp cells; 0.25, 0.5 and 1 μmol of Gln for HGC27 and HGC27/ddp cells; 0.5 and 1 μmol of Gln for AGS and AGS/ddp cells), the maximum number of sensitive cells did not increase, but the maximum number of resistant cells increased significantly in our Gln-restriction model (Fig. 1B,C and D). These results were confirmed using a CFSE assay in HeLa and HeLa/ddp cell lines (Fig. 2A). Interestingly, cell growth was not influenced by the different Gln concentrations used in our study, which were tested in our model when sufficient Gln was supplied (medium was changed daily) (Fig. 2B). Furthermore, increased Gln consumption in all three ddp-resistant cell lines was confirmed by comparing the concentration of Gln in the culture medium (Fig. 2C). These results indicated that the differences in the maximum cell number were due to exhaustion of Gln and not different Gln concentrations in our Gln-restriction model. We further demonstrated that all three sensitive cell lines produced more cells than the corresponding resistant cell lines when cultured with the same amount of Gln in our model (Fig. 2D). HeLa cells growing from 0.1 million to 0.8 million cells required only 0.5 μmol Gln (Fig. 1B), but HeLa/ddp cells required nearly 2 μmol Gln to reach the same number, indicating that Gln consumption in resistant cells was nearly 3- to 4-fold greater than that in sensitive cells. Our results indicated that Gln consumption in resistant cells was much greater than that in sensitive cells.

### Antioxidant capacity is enhanced in ddp-resistant cells and directly related to the presence of Gln

**Figure 3:**
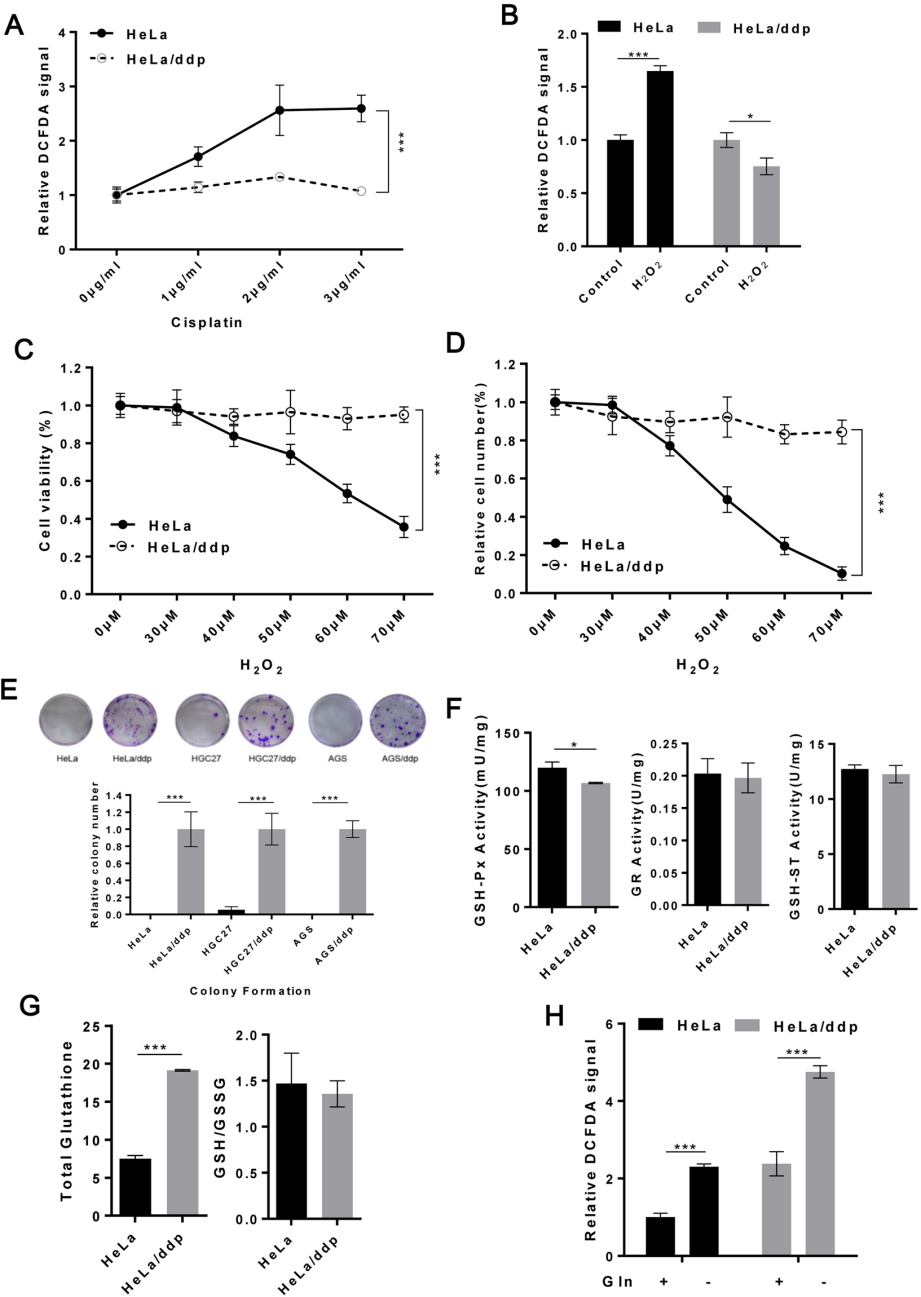
Ddp-resistant cells were resistant to oxidative stress, which was related to the availability of Gln. (**A**) Relative ROS levels in HeLa and HeLa/ddp cells treated with ddp for 24 h. (**B**) Relative ROS levels in HeLa and HeLa/ddp cells treated with H_2_O_2_ for 24 h. (**C** and **D**) Relative cell viability and cell numbers for HeLa and HeLa/ddp cells treated with H_2_O_2_ for 72 h. (**E**) Relative clonogenic growth of six cell lines in the presence of H_2_O_2_ (20 μM for HeLa and HeLa/ddp, 10 μM for HGC27, HGC27/ddp, AGS and AGS/ddp). (**F** and **G**) GSH-ST activity, GSH-Px activity, GR activity, total glutathione and GSH/GSSG ratio in HeLa and HeLa/ddp cells. (**H**) Relative ROS levels in HeLa and HeLa/ddp cells cultured with corresponding medium for 24 h. The error bars represent the s.d. of triplicate wells of a representative experiment.

One of the toxic effects of ddp induces ROS generation in the cytoplasm, which in turn exacerbates ddp-induced cell damage. In our study, ddp induced intracellular ROS accumulation in sensitive cells but not in resistant cells (Fig. 3A). A similar result was also observed after H_2_O_2_ treatment (Fig. 3B). We also compared the growth-suppressive effect of H_2_O_2_ on both types of cell lines and found that resistant cells were markedly more resistant to oxidative stress (Fig. 3C and D), a finding also confirmed by a colony formation assay (Fig. 3E). We observed no difference in glutathione S-transferase (GSH-ST) or glutathione reductase (GR) activity between the two cell populations, although glutathione peroxidase (GSH-Px) activity was slightly decreased in resistant cells (Fig. 3F). These findings suggested that the enhanced antioxidant capacity of resistant cells did not depend on these three antioxidant enzymes. In a subsequent experiment, we found approximately 3-fold higher cellular total GSH levels in resistant cells than in sensitive cells (Fig. 3G), which was consistent with findings in human ovarian cancer^25^. Although no difference was observed in the ratio of reduced-to-oxidized GSH (GSH:GSSG) (Fig. 3G), resistant cells still exhibited higher GSH levels compared to sensitive cells. Given that resistant cells consume substantially more Gln to sustain growth and that Gln can participate in the intracellular redox balance, we examined whether Gln affected the redox balance in both cell lines. Gln deprivation markedly increased ROS accumulation in both cell lines (Fig. 3H), suggesting that enhanced antioxidant capacity might be related to excess Gln consumption in the resistant cell lines.

### Gln catabolism is enhanced in resistant cells, which maintains cellular redox homeostasis

**Figure 4:**
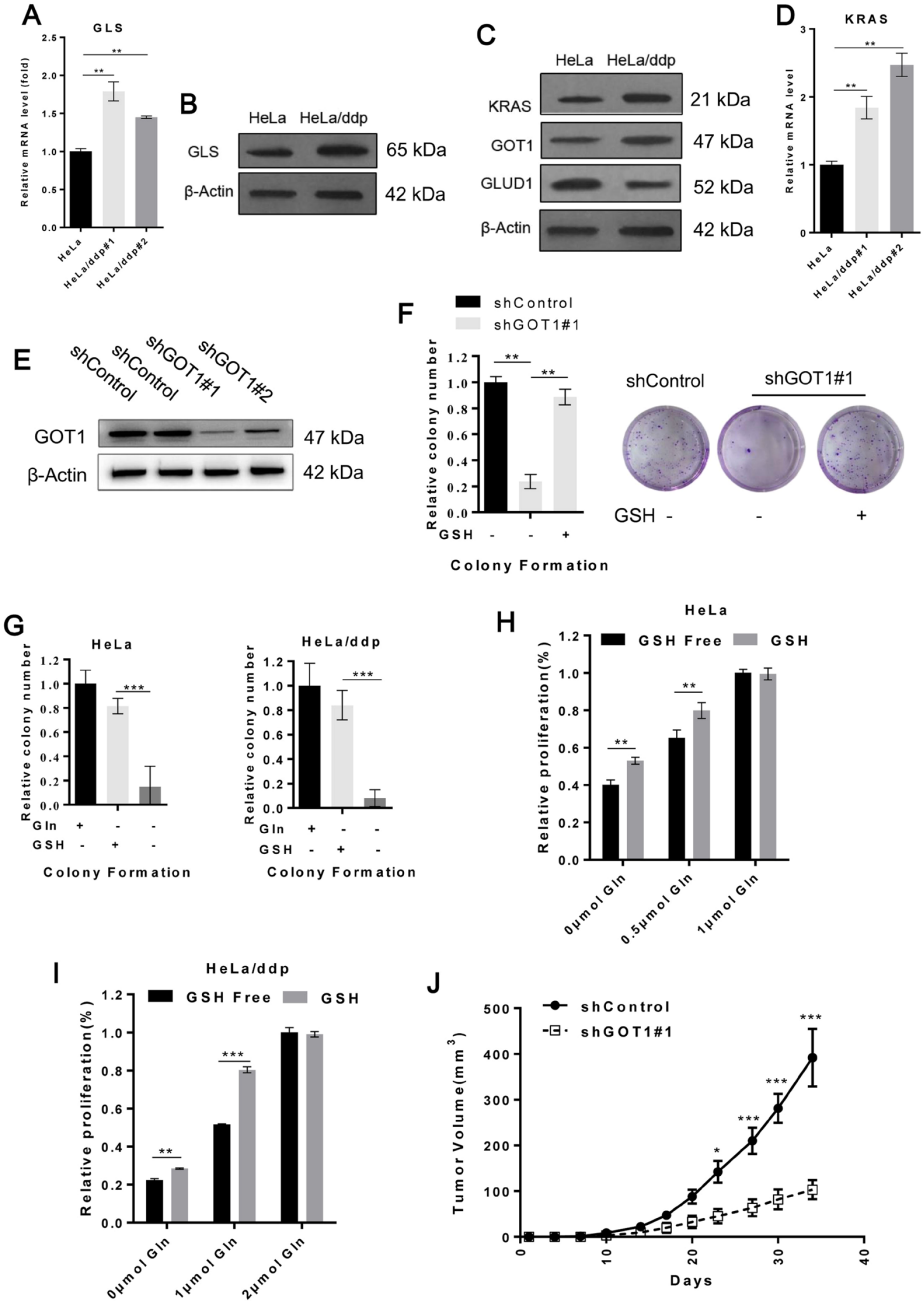
Gln catabolism was enhanced in resistant cells to sustain cellular redox homeostasis. (**A**) qRT-PCR shows the relative expression of GLS mRNA in HeLa and HeLa/ddp cells. (**B**) Western blot analysis for GLS and β-Actin expression in HeLa and HeLa/ddp cells. **(C**) Western blot analysis of KRAS, GOT1, GLUD1 and β-Actin expression in HeLa and HeLa/ddp cells. (**D**) qRT-PCR shows the relative expression of KRAS mRNA in HeLa and HeLa/ddp cells. (**E**) The expression of GOT1 and β-Actin was determined by Western blot analysis in HeLa/ddp cells transfected with negative control shRNA or two independent shRNAs targeting GOT1 for 48 h. (**F**) HeLa/ddp cells were transfected with a negative control shRNA or shRNA targeting GOT1 for 48 h, and then the cells were seeded in 6-well plates. The medium was replaced the following day with Gln-free medium, and GSH (4 mM) was added to the medium following Gln withdrawal. (**G**) Relative clonogenic growth of HeLa and HeLa/ddp cells. GSH (4 mM) was added to the medium following Gln withdrawal. (**H** and **I**) Ralative proliferation of HeLa and HeLa/ddp cells determined by CFSE assay; the medium was replaced with corresponding medium and supplemented with GSH (4 mM) the following day. The error bars represent the s.d. of triplicate wells of a representative experiment (**A**–**I**). (**J**) Xenograft growth of HeLa/ddp cells expressing a control shRNA or a shRNA targeting GOT1 in mice (n = 5). The error bars represent the s.e.m.

We observed that the expression of glutaminase (GLS) was increased in ddp-resistant cells, indicating that Gln catabolism was accelerated (Fig. 4A and B). We analyzed the levels of KRAS pathway proteins to determine whether KRAS-mediated metabolic reprogramming, which increases glutamic-oxaloacetic transaminase 1 (GOT1) expression and decreases glutamate dehydrogenase (GLUD1) expression, accounts for the increased Gln consumption of resistant cells. The results of western blot analysis revealed elevated KRAS and GOT1 levels and decreased GLUD1 levels in resistant cells (Fig. 4C), and the level of KRAS mRNA was also significantly increased in ddp-resistant cells. By using lentiviral shRNA of GOT1 (Fig. 4E), we found that GOT1 knock down significantly inhibited the growth of ddp-resistant cells, which could be rescued by GSH (Fig. 4F). Together, these results suggested that the KRAS-mediated metabolic reprogramming pathway was up-regulated in resistant cells.

To further confirm that Gln was used to maintain redox homeostasis, we treated both cell lines grown in Gln-free complete medium with a cell-permeable GSH analogue. GSH treatment dramatically rescued clonogenic growth in both cell lines following Gln deprivation (Fig. 4G and Supplementary Fig. S1B), which was also confirmed by CFSE assay (Fig. 4H and I; Supplementary Fig. S2A and S2B). GSH decreased Gln consumption in both cell lines in our Gln-restriction model (Supplementary Fig. S3A and S3B), and the growth limitations of both cell lines could not be increased in the presence of sufficient Gln, suggesting that GSH could only rescue the growth limitation caused by insufficient Gln (Fig. 4H and I). As further confirmation of the importance of this pathway in ddp-resistant cells, we suppressed GOT1 expression using lentiviral shRNA in HeLa/ddp cells and assessed their growth *in vivo*. Consistent with our *in vitro* results, GOT1 knockdown significantly decreased ddp-resistant tumor growth *in vivo* (Fig. 4J). In summary, Gln catabolism in resistant cells was increased through KRAS-mediated metabolic reprogramming and played an important role in maintaining the redox state.

### Rapid Gln catabolism is indispensable for maintaining ddp resistance but unnecessary for ddp-resistant cell growth in the absence of ddp

**Figure 5:**
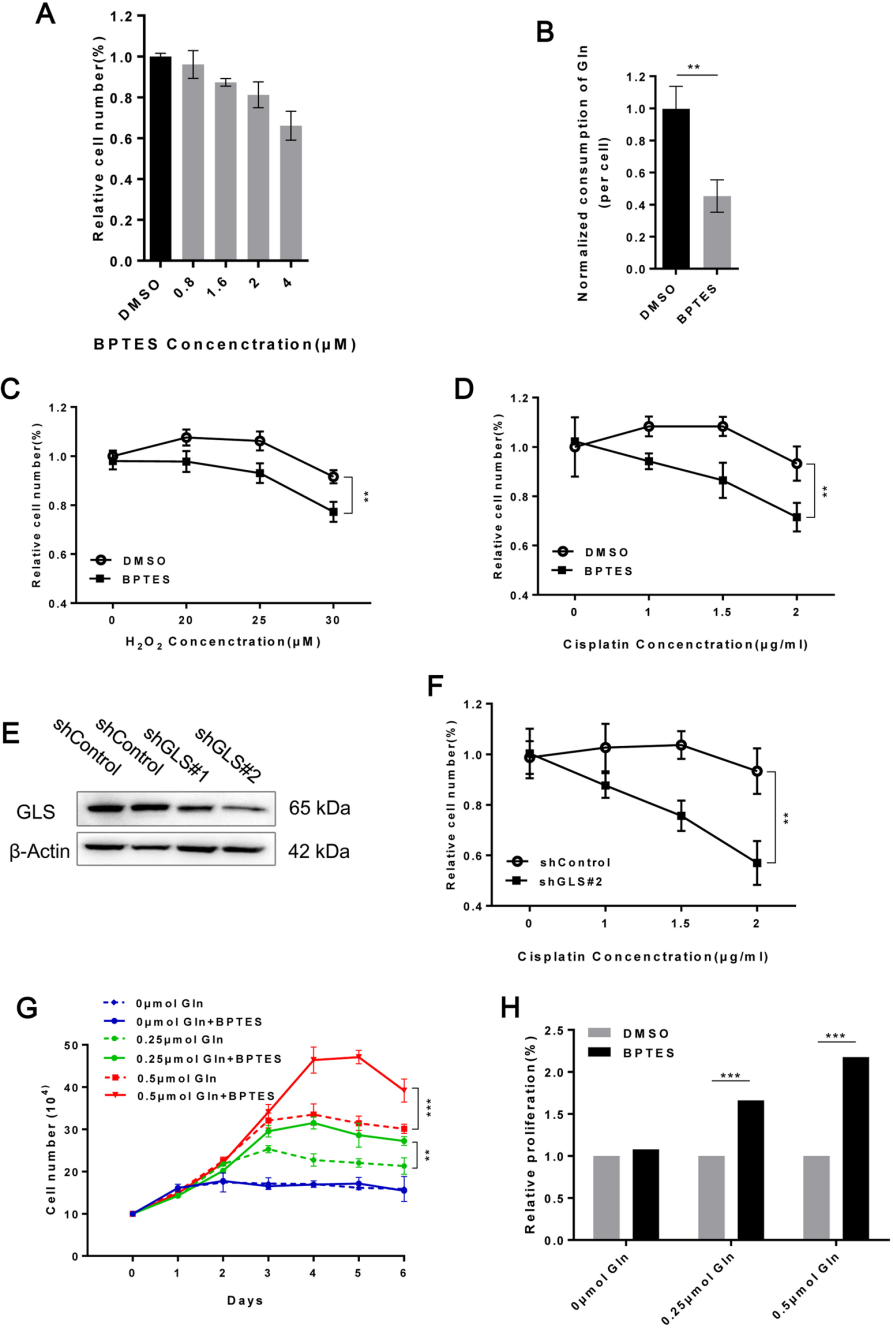
Rapid Gln consumption is a growth burden for resistant cells but indispensable for drug resistance. (**A**) Relative number of HeLa/ddp cells treated with the indicated concentrations of the GLS inhibitor (BPTES) for 72 h. (**B**) Quantification of Gln consumption in HeLa/ddp cells after partial suppression of GLS activity (0.8 μM BPTES) for 24 h. (**C** and **D**) Relative proliferation of HeLa/ddp cells treated with increasing concentrations of H_2_O_2_ and ddp after partial suppression of GLS activity (0.8 μM BPTES) for 72 h. (**E**) The expression of GLS and β-Actin was determined by Western blot analysis in HeLa/ddp cells transfected with a negative control shRNA or two independent shRNAs targeting GLS for 48 h. (**F**) HeLa/ddp cells were transfected with a negative control shRNA or shRNA targeting GLS for 48 h, and then the cells were treated with ddp for 72 h. (**G**) Growth of HeLa/ddp cells after partial suppression of GLS activity (0.8 μM BPTES). (**H**) Relative proliferation of HeLa/ddp cells after partial suppression of GLS activity (0.8 μM BPTES) was determined using CFSE assay. The error bars represent the s.d. of triplicate wells of a representative experiment.

GLS, which catalyzes the conversion of Gln to glutamate, is a key enzyme in the initiation of Gln catabolism. We found that GLS overexpression mediated the rapid Gln catabolism in resistant cells (Fig. 4A and B). Next, we investigated the impact of GLS overexpression on ddp resistance and cell growth. Appropriate GLS suppression by BPTES (0.8 μM) could significantly reduce the Gln consumption of resistant cells without impacting cell growth (Fig. 5A and B). Then, we observed that ddp-resistant cells were more sensitive to H_2_O_2_ and ddp once Gln catabolism was partially blocked with 0.8 μM BPTES (Fig. 5C and D). By using lentiviral shRNA of GLS (Fig. 5E), we also confirmed that ddp-resistant cells were more sensitive to ddp after partial GLS knock down (Fig. 5F). These results indicated that rapid Gln catabolism provided additional reduced hydrions to generate GSH, which subsequently rapidly scavenged ROS and mediated ddp resistance. Whether rapid Gln catabolism affects the growth of ddp-resistant cells was still unknown. Therefore, we next evaluated the effects of partial GLS suppression on the maximum cell number achieved in our Gln-restriction model. Interestingly, the maximum number of ddp-resistant cells increased significantly under the same Gln conditions upon partial GLS suppression with 0.8 μM BPTES (Fig. 5G); this result was confirmed in the CFSE assay (Fig. 5H and Supplementary Fig. S3C). These findings revealed that more ddp-resistant cells could be produced if we slowed down Gln catabolism under the same Gln conditions. Taken together, our results indicated that the crucial defense mechanism in resistant cells under drug pressure became unnecessary and served as a fitness deficit for cell growth in the absence of ddp.

## Discussion

Many theories suggest that drug-resistant cells are less fit than drug-sensitive cells^12,14,16^, but comprehensive and direct experimental evidence is lacking. To uncover the relationship between drug resistance and cell fitness in this study, we established three ddp-resistant cell lines by exposing parental cells to gradually increasing concentrations of ddp. For the first time, our work showed that ddp-resistant cells consumed substantially more Gln to support their growth by KRAS-mediated metabolic reprogramming in multiple ddp-resistant cell lines, but the extra Gln consumption became a fitness deficit in ddp-resistant cells in the absence of the drug. Increased antioxidant capacity for ddp resistance is a double-edged sword; although it enables resistant cells to survive cytotoxic treatment, it also slows the growth of ddp-resistant cells by excess Gln consumption.

In our study, ddp-resistant cell growth could be efficiently inhibited by suppressing the expression of GOT1 alone instead of GOT1 suppression together with cisplatin, indicating that GOT1 plays a critical role in ddp-resistant cell growth. Our work suggests that GOT1 may serve as a potential target for the development of anti-ddp-resistant cell therapeutics, and GOT1 inhibition may be a novel strategy to target a subset of patients with tumors that are resistant to ddp.

Multiple mechanisms of ddp-induced cytotoxicity and anti-proliferation exist in tumor cells^19^, and accordingly, a number of defense mechanisms may exist in ddp-resistant cells. ROS induced by ddp can directly or indirectly exacerbate ddp-induced cell damage. Our results confirmed that ddp-resistant cells were also resistant to oxidative stress, which was achieved by rapid Gln consumption even in the absence of the drug. However, limited Gln was available for tumor growth, and HeLa cells could produce more descendants in the same amount of Gln, which indicated that the proliferation of HeLa cells was more efficient than that of HeLa/ddp cells. In this case, evolution would favor more efficient proliferation.

Adaptive therapy, in which treatment is continuously adjusted according to the tumor response to gain a fixed tumor volume, was first proposed by Gatenby, and the principle was to control tumor development by permitting a certain amount of chemosensitive cells to survive so that they could inhibit the growth of less fit but chemoresistant cells^13^. Researchers have successfully used adaptive therapy to control tumor growth in different preclinical models of breast cancer^15^. Compared with traditional high dose treatment, adaptive therapy results in substantially prolonged survival.

In summary, our study revealed that to sustain increased antioxidant capacity, which mediated drug resistance, ddp-resistant cells must maintain rapid Gln catabolism through KRAS-mediated metabolic reprogramming, even in the absence of ddp. Consequently, ddp-resistant cells consume more Gln to support their growth, which becomes a fitness deficit in ddp-free conditions. This is the first study showing that ddp-resistant cells are less fit than their parental cells in the absence of the drug due to the ddp-resistance mechanism. Our results provide direct evidence for the development of a ddp-treatment strategy following the principle of adaptive therapy, which may significantly increase patient survival time.

## Materials and Methods

### Cell culture

HeLa, HGC27, and AGS cells were purchased from the Type Culture Collection of the Chinese Academy of Sciences, Shanghai, China. HeLa/ddp, HGC27/ddp, and AGS/ddp cell lines were established by continuous exposure of the parental cells to gradually increasing concentrations of ddp (P4394, Sigma) as described in our previous study^18^, and the IC_50_ values of the six cell lines were determined using CCK8 assays (Supplementary Fig. S1A). All cell lines were authenticated by short tandem repeat profiling analysis in 2014.

### Cell culture medium

All cell lines were cultured in RPMI 1640 (Invitrogen) supplemented with 10% FBS (Biological Industries, BI) and 1% penicillin-streptomycin (50 µnits/ml, 50 µg/ml, Invitrogen) under a 5% CO_2_ environment. All media used in our experiments were supplemented with 10% FBS and 1% penicillin-streptomycin. Media with different Gln concentrations (0.5 and 1 mM) were prepared by diluting normal RPMI 1640 (2 mM Gln) with Gln free RPMI 1640 (Invitrogen), and 4 mM Gln RPMI 1640 was prepared by the addition of 1% 200 mM Gln solution (Invitrogen) to normal RPMI 1640. The total amount of Gln was limited by fixing the volume of media (0.5, 1, 2 and 4 mM Gln) in 24-well plates at 0.5 ml/well.

### Cell growth analysis under a Gln-restriction model

Cells were plated in 24-well plates at 1 × 10^5^ (HeLa) or 5 × 10^4^ (HGC27 and AGS) cells per well in 0.5 ml of media and grown for 24 h, at which time the medium was changed to contain different Gln concentrations (0.5 ml). The medium was not altered throughout the course of the experiment. At the indicated time points, cells in triplicate wells were trypsinized and counted using a hand-held automated cell counter (Scepter 2.0, Millipore) for six days, allowing tumor cells sufficient time to exhaust the Gln in the limited medium in our model.

### CFSE assay

Cells were labeled with carboxyfluorescein diacetate succinimidyl ester (CFSE, eBioscience) according to the manufacturer’s instructions. The labeled cells were cultured or treated as required and analyzed by flow cytometry (BD Bioscience) at the end of the experiment. Proliferation was calculated based on the following methods: *C*_0_, *C*_1_ and *C*_2_ represent the initial cell count, control group cell count and experimental group cell count, respectively, at the indicated time points. *n*_1_ and *n*_2_ represent the average population doublings for the control and experimental groups, respectively, at the indicated time points. *FI*_0_, *FI*_1_ and *FI*_2_ represent the initial average fluorescence intensities and the average fluorescence intensities of the control and experimental groups, respectively, at the indicated time points.

$${\rm{Relative}}\,{\rm{proliferation}}=\frac{{C}_{2}}{{C}_{1}}=\frac{{C}_{0}{2}^{{n}_{2}}\,}{{C}_{0}{2}^{{n}_{1}}\,}=\frac{{C}_{0}F{I}_{0}/F{I}_{2}\,}{{C}_{0}F{I}_{0}/F{I}_{1}}=\frac{F{I}_{1}}{F{I}_{2}}$$

### Cell viability and proliferation analysis

HeLa and HeLa/ddp cell lines were plated onto 24-well plates at 1.5 × 10^4^ cells per well in 0.5 ml of growth media. On the following day, the growth medium was replaced with medium containing H_2_O_2_ and/or GLS inhibitors. Parallel plates were analyzed after 3 or 6 days by CCK8 (Dojindo) according to the manufacturer’s instructions or by crystal violet staining analysis. For the crystal violet staining assay, cells were fixed in 10% formalin and stained with 0.1% crystal violet. The dye was extracted with 10% acetic acid, and the relative proliferation was measured by absorbance at 490 nm.

### Analysis of glutamine metabolism

Cells were plated in 6-well plates at 4 × 10^5^ cells per well, and the medium was changed to complete medium (2 mM Gln) on the following day. The cell number was counted, and medium was collected and analyzed using the Glutamine and Glutamate Determination Kit (GLN1, Sigma) after 24 hours. Gln consumption was compared with that in medium without cells under the same condition and normalized to the cell number.

### Colony formation assay

For the colony formation assay, cells were plated in 6-well plates at 500–1,000 cells per well in 3 ml of media. The media were not altered throughout the course of the experiment. After 10–14 days, colonies were fixed in methanol and stained with 0.5% crystal violet. Colonies of more than 50 cells were counted under a microscope.

### Measurement of GSH, GSH/GSSG, GSH-ST, GSH-Px and GR activities

Intracellular levels of GSH and GSH/GSSG, GSH-ST activity, GSH-Px activity and GR activity were determined using the GSH and GSSG Assay Kits (Beyotime), Glutathione S-transferase Assay Kit (KeyGEN BioTECH), Cellular Glutathione Peroxidase Assay Kit (Beyotime) and Glutathione Reductase Assay Kit (Beyotime), respectively, according to the manufacturer’s instructions.

### ROS quantification

Cells were treated as desired and incubated with 5 μM 2′,7′-dichlorofluorescein diacetate (DCFDA, Sigma) for 25 min. Cells were washed two times with PBS to remove excess DCFDA. Labeled cells were trypsinized and resuspended in PBS. The mean fluorescence intensity was determined by flow cytometry.

### RNA extraction and real-time quantitative PCR (qRT-PCR)

Total RNA was extracted using TRIzol reagent (Invitrogen), and reverse transcription was performed with PrimeScript^TM^ RT Master Mix (Takara). qRT-PCR was performed using SYBR Premix Ex Taq reagents (Takara). Primers: GLS, 5′-AGGGTCTGTTACCTAGCTTGG-3′ (forward) and 5′-ACGTTCGCAATCC-TGTAGATTT-3′ (reverse); β-actin, 5′-AGCGAGCATCCCCCAAAGTT-3´ (forward) and 5′-GGGCACGAAGGCTCATCATT-3′ (reverse). The expression of β-actin was measured as an internal control.

### Lentiviral-mediated shRNA transfection

Scrambled control shRNA, GOT1 shRNA and GLS shRNA were synthesized by GenePharma (Shanghai, China). These shRNA constructs were then packed into lentiviruses and applied for infection of HeLa/ddp cells. The sequences for each shRNA were as follows: shGOT1#1 (GCGTTGGTACAATGGAACAAA), shGOT1#2 (GCTAATGACAATAGCCTAAAT), shGLS#1 (GCACAGACATGGTTG GTATAT) and shGLS#2 (GCCCTGAAGCAGTTCGAAATA).

### Western blot analysis

Cells were washed with cold PBS and lysed in RIPA buffer supplemented with a protease inhibitor cocktail. Protein lysates were separated by SDS-PAGE and transferred to polyvinylidene fluoride membranes. The membranes were blocked in TBST (Tris-buffered saline containing 0.1% Tween-20) containing 5% non-fat dry milk or bovine serum albumin before overnight incubation with the desired primary antibodies at the dilution recommended by the instructions. The membranes were washed with TBST, followed by exposure to the appropriate horseradish peroxidase (HRP)-conjugated secondary antibody (1:5000 dilutions). Then, the membranes were visualized on Kodak X-ray film using Millipore Immobilon Western Chemiluminescent HRP Substrate, or signals generated by enhanced chemiluminescence (Millipore) were recorded with a CCD camera (CLINX, Shanghai, USA). The following antibodies were used: KRAS (F234, Santa Cruz), GOT1 (ab111501, Abcam), GLUD1 (ab166618, Abcam), GLS (ab156876, Abcam) and β-Actin (A5441, Sigma).

### Xenograft experiments

Female Nu/Nu mice were purchased from Vital River Laboratories. For subcutaneous xenografts, HeLa/ddp cells were infected with control lentiviral shRNA or lentiviral shRNA targeting GOT1. Then, 3 × 10^6^ cells were suspended in 0.1 ml of 50% Matrigel (BD Biosciences) solution in RPMI 1640 and injected subcutaneously into the lower flanks of 4-week-old mice. Tumor length and width were monitored bi-weekly using electronic calipers. Tumor volumes were calculated using the following formula: 1/2 × length × width2. All xenograft experiments were approved by the Ethics Review Committee for Animal Experimentation at Drum Tower Hospital (Nanjing, China). All animal procedures were performed in compliance with guidelines set by the Animal Care Committee, and all efforts were made to reduce potential pain and discomfort in the animals.

### Statistical analyses

All experiments were performed in triplicate. Data are presented as the mean ± SD. Differences between treatments were evaluated by ANOVA or Student’s t test. Differences were considered significant if P < 0.05 (*P < 0.05; **P < 0.01; and ***P < 0.001). All statistical analyses were performed using Prism 6.0.

### Availability of materials and data

All data generated or analysed during this study are included in this published article (and its Supplementary Information files).

## Electronic supplementary material

Supplementary information
