# Supplementary material for: Increased Glutamine Consumption in Cisplatin-Resistant Cells Has a Negative Impact on Cell Growth
Source: Sci Rep. 2018 Mar 6;8:4067. doi: 10.1038/s41598-018-21831-x (PMC5840399; doi:10.1038/s41598-018-21831-x)
Supplement: Supplementary file 1 — Supplementary information [file 41598_2018_21831_MOESM1_ESM.pdf]

Supplementary Information

Increased Glutamine Consumption in Cisplatin-Resistant Cells Has a Negative Impact on Cell Growth

Authors and Affiliations: Guihua Duan[a,b], Mengyue Shi[a], Lijuan Xie[c], Mingcui Xu[a], Yun Wang[a], Hongli Yan[d], Yuzheng Zhuge[a] and Xiaoping Zou[a]

a Department of Gastroenterology, Drum Tower Hospital, Medical School of Nanjing University, Nanjing 210008, Jiangsu Province, China

b Department of Gastroenterology, The First People's Hospital of Yunnan Province, The Affiliated Hospital of Kunming University of Science and Technology, Kunming 650032, China.

c Department of Special Medical Treatment, First Affiliated Hospital of Kunming Medical University, Kunming 650332, China

d Department of Laboratory Medicine, Changhai Hospital, Second Military Medical University, Shanghai 200433, China

Figure S1. (A) IC50 values for HeLa, HeLa/ddp, HGC27, HGC27/ddp, AGS and AGS/ddp cells. The error bars represent the s.d. of triplicate wells from a representative experiment. (B) Representative images of the colony formation assay depicted in Figure 4G

Figure S2. (A) Representative images of the CFSE assay depicted in Figure 4H. (B) Representative images of the CFSE assay depicted in Figure 4I.

Figure S3. (A) and (B) Growth of HeLa and HeLa/ddp cells in medium supplemented with GSH, medium was replaced with corresponding medium and supplemented with GSH (4 mM) the following day. (C) Representative images of the CFSE assay depicted in Figure 5H. The error bars represent the s.d. of triplicate wells from a representative experiment.

Figure S4. Full-length Western blots images of main Figure 4E and 5E.

Supplemental Figure 1

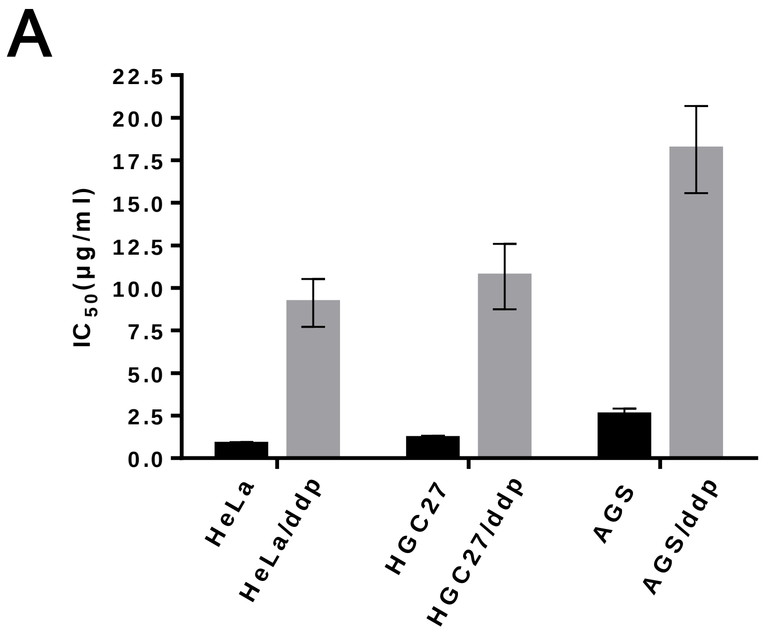

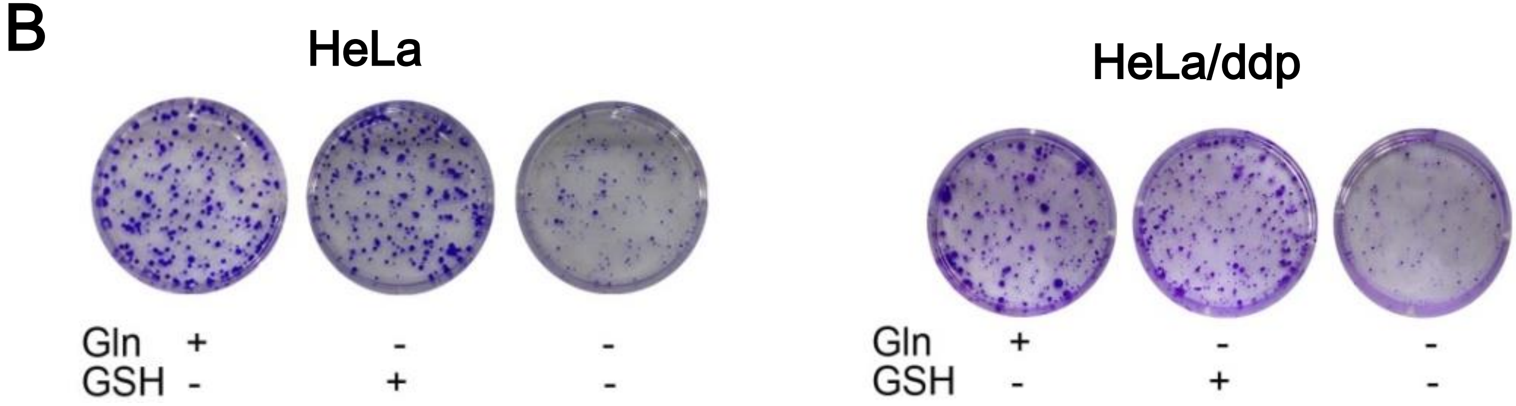

Supplemental Figure 2

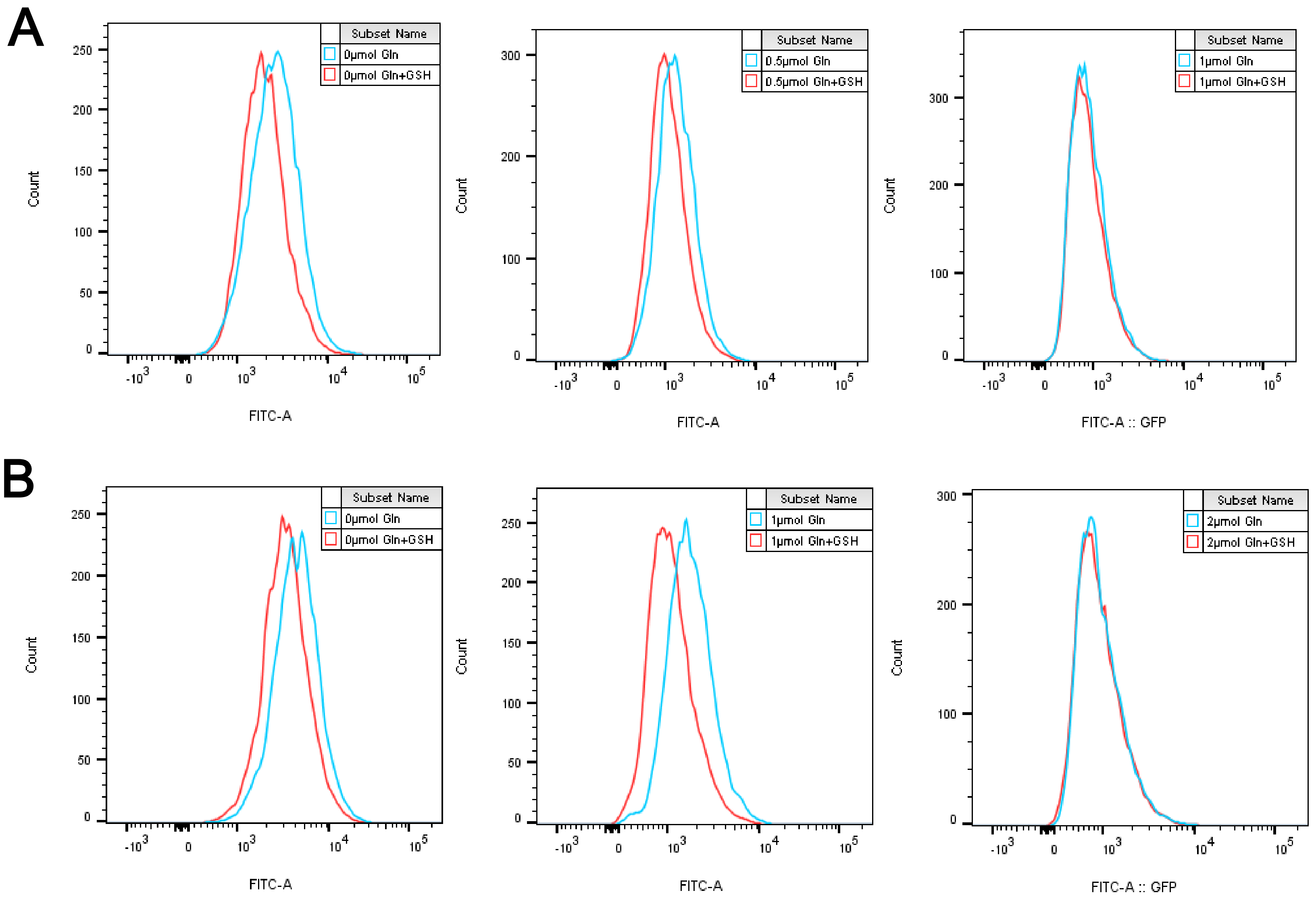

Supplemental Figure 3

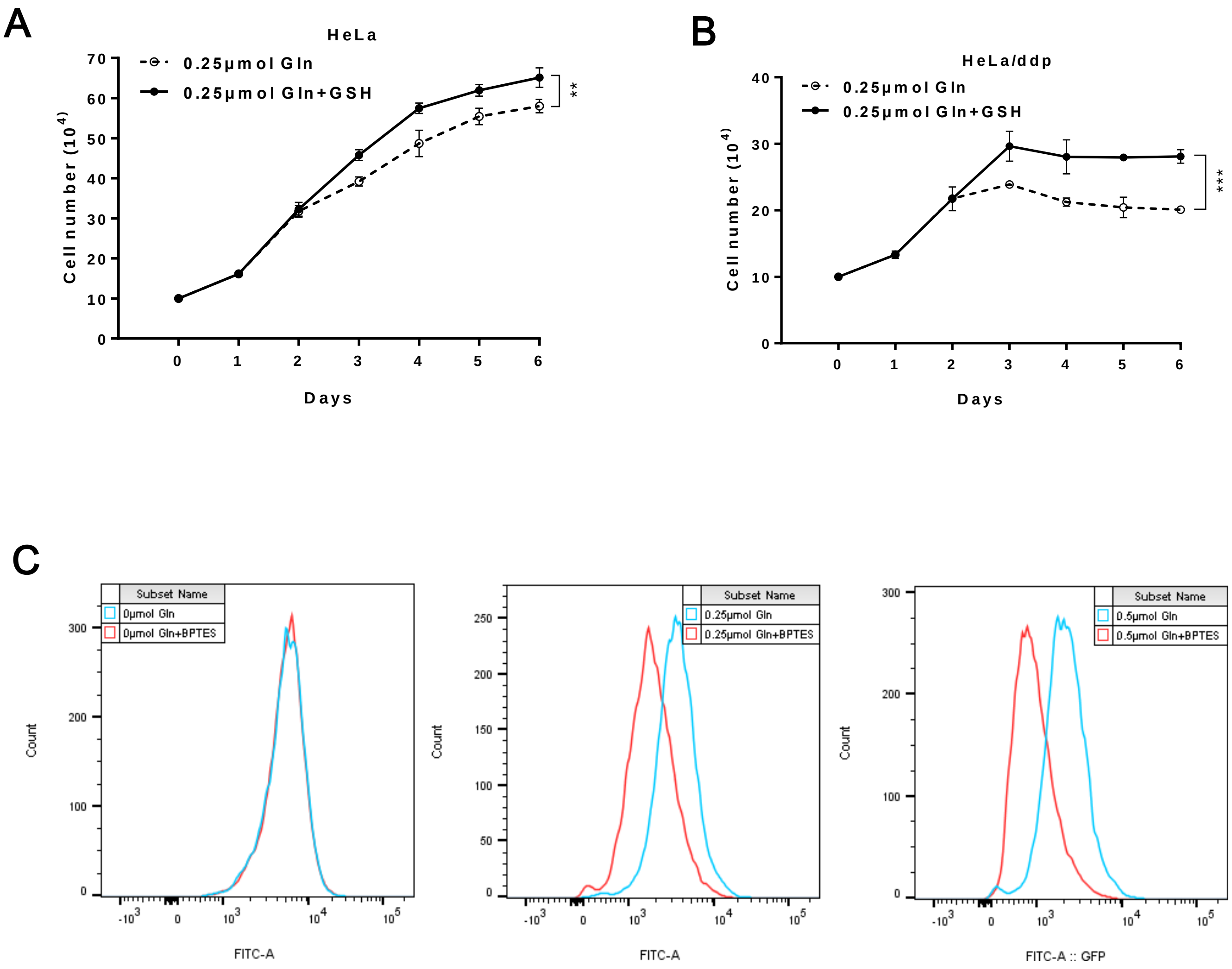

Supplemental Figure 4

Figure 4E

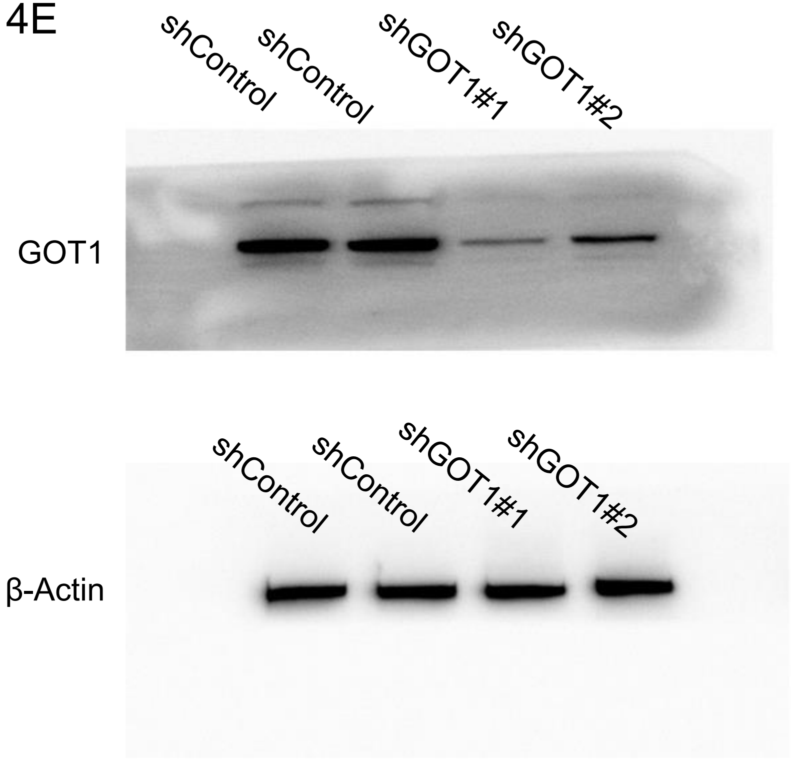

Figure 5E

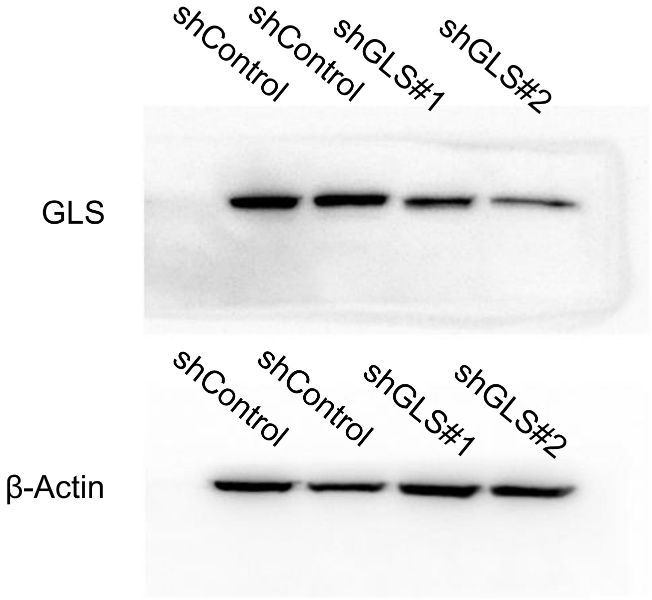

Full-length Western blots images of main Figure 4E and 5E
